# Exploring the limits of generalized dissociative amnesia: A case report

**DOI:** 10.1192/j.eurpsy.2023.2340

**Published:** 2023-07-19

**Authors:** C. I. Varlam, V. Dionisie, R. Movileanu, G. Andrișca, A. Roșu, M. Manea

**Affiliations:** 4th Department, “Prof. Dr. Alexandru Obregia” Clinical Hospital of Psychiatry, Bucharest, Romania

## Abstract

**Introduction:**

Dissociative amnesia (DA), one of several dissociative disorders, is characterized by the inability to recall autobiographical information that is inconsistent with normal forgetting. Generalized amnesia is a rare subtype of amnesia distinguished by the acute onset of complete loss of memory for one’s life history, in which the patients may lose semantic knowledge, procedural knowledge or personal identity.

**Objectives:**

The objective of this paper is to highlight that the diagnosis of generalized DA can be controversial and a comprehensive history, as well as collateral information, are essential for an accurate diagnosis.

**Methods:**

We present the case of a 37-year-old female, with no premorbid medical illness and one year psychiatric history which was admitted to our clinic for severe deficits of the memory and attention functions, emotional lability, social withdrawal, strong socio-professional dysfunctionality, altered behavior marked by the subjective changes of memory and thinking processes, affective ambivalence towards parents, mixed insomnia. The hetero-anamnesis revealed that our patient presented two fugues during the last 4 months. During her mental status evaluation, she showed temporal and spatial orientation, demonstrative attitude, spontaneous speech centered on her mental suffering, euthymic disposition, delusional ideas with somatic content, intermittent and inconstant facial motor stereotypes.

**Results:**

Multiple neurological examinations were performed, all being within normal limits. The magnetic resonance imaging of the brain identified an enlarged adenohypophysis and a possible microaneurysm that do not correlate with the symptoms. The endocrinological investigations invalidated the suspicion of acromegaly. The psychological examination suggested the tendency to mask less acceptable feelings, inadequacy, rigidity, the presence of conflicts of a sexual nature, regression to an infantile stage, with a deficit of concentrated attention. The emergent symptoms and signs were resistant, failed to resolve with antidepressant and antipsychotic medication and continued to persist across all settings. Corroborating evidence, we established the diagnosis of DA.

**Conclusions:**

DA represents a controversial diagnostic entity that incorporates elements of psychogenic fugue states, repressed memory, traumatic amnesia, and conversion. Some clues in the history such as psychological traumas can support a diagnosis of DA rather than medical causes. As with most other psychiatric disorders, it is important to rule out organic causes first before considering psychiatric etiologies. A thorough sequential history and collateral information are key components in effective diagnosis and management of this condition. In the absence of a favorable response to psychotropic drugs, psychotherapy represents the best treatment approach for DA.

**Disclosure of Interest:**

None Declared

